# Bacterial floc mediated rapid streamer formation in creeping flows

**DOI:** 10.1038/srep13070

**Published:** 2015-08-17

**Authors:** Mahtab Hassanpourfard, Zahra Nikakhtari, Ranajay Ghosh, Siddhartha Das, Thomas Thundat, Yang Liu, Aloke Kumar

**Affiliations:** 1 Department of Chemical and Materials Engineering, University of Alberta, Edmonton, Canada; 2 Department of Mechanical Engineering, University of Alberta, Edmonton, Canada; 3 Department of Mechanical and Industrial Engineering, Northeastern University, Boston MA 02115, USA; 4 Department of Mechanical Engineering, University of Maryland, College Park, MD 20742, USA; 5 Department of Civil and Environmental Engineering, University of Alberta, Edmonton, Canada

## Abstract

One of the central puzzles concerning the interaction of low Reynolds number 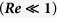 fluid transport with bacterial biomass is the formation of filamentous structures called streamers. In this manuscript, we report our discovery of a new kind of low *Re* bacterial streamers, which appear from pre-formed bacterial flocs. In sharp contrast to the biofilm-mediated streamers, these streamers form over extremely small timescales (less than a second). Our experiments, carried out in a microchannel with micropillars rely on fluorescence microscopy techniques to illustrate that floc-mediated streamers form when a freely-moving floc adheres to the micropillar wall and gets rapidly sheared by the background flow. We also show that at their inception the deformation of the flocs is dominated by recoverable large strains indicating significant elasticity. These strains subsequently increase tremendously to produce filamentous streamers. Interestingly, we find that these fully formed streamers are not static structures and show viscous response at time scales larger than their formation time scales. Finally, we show that such novel streamer formation can lead to rapid clogging of microfluidic devices.

In their natural state bacteria can be found in either disparate planktonic forms or living in tight knit communities such as flocs, mats, pellicles or biofilms^1^,^2^,^3^. The latter, aggregative modes of bacterial growth are characterized by cells embedded in a matrix, usually of self-produced extracellular polymeric substances (EPS) composed of long-chain biomolecules such as polysaccharides, nucleic acids and lipids^1^,^4^,^5^,^6^,^7^. This composite soft matter, consisting of bacteria and EPS, has been attracting intense scrutiny due to a complex interplay between material behavior and the underlying life processes brought about by large deformation even at very low Reynold’s number (*Re*); for example, in the case of filamentous bacterial streamers generated from bacterial biofilms^8^,^9^,^10^,^11^.

Streamers are so named due to their distinguishing filamentous morphology. They have been reported in systems with sustained hydrodynamic flows^9^. These slender bacterial aggregates are typically tethered at one or both ends to solid surfaces, while the rest of the structure is suspended in a liquid environment. Bacterial streamers have been observed to form both in high^12^,^13^, and in low Reynolds number conditions 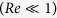
10,11,14,15,16. Streamer formation in low *Re* transport is of significant technological and biomedical interest due to relevance to a wide variety of critical operating scenarios including clogging of biomedical devices such as heart stents, catheters, porous media and water filtration systems^8^,^11^,^17^. Rusconi *et al.*^10^, while studying the effect of curved channel geometries using a microfluidic device, found that *Pseudomonas aeruginosa* formed streamers in the curved sections of the microchannels. Drescher *et al.*^8^ later showed that streamer formation in microfluidic devices with curved sections can lead to catastrophic clogging. Valiei *et al.*^11^ used a microfluidic device with micropillars, and studied biofilm formation by *Pseudomonas fluorescens*. They found that, in a certain flow regimes, the bacteria formed extensive streamers resulting in a web-like network between the different pillars. A commonality between the reports by Valiei *et al.*^11^, Rusconi *et al.*^10^ and Drescher *et al.*^8^ is that the streamers appeared far later than the biofilms, and the corresponding streamer formation time-scale, *t*_*s*_, was of the order of hours from the beginning of the flow. Das and Kumar^18^ have recently proposed that in such instances, where the streamer formation time-scale far exceeded the relaxation time-scale of biofilms, streamers appeared from a highly viscous state of the intrinsically viscoelastic biofilms. In contrast to these studies, some other experiments conducted under apparently similar creeping flow conditions reported much smaller *t*_*s*_ values (*t*_*s*_ ~ minutes)^15^,^16^ (see Table 1). Kim *et al.*^19^ have reported even smaller streamer formation time scales for the bacterium *Staphylococcus aureus*, though this was achieved by first coating the channel walls of the microfluidic device with human plasma. Such large variation in streamer formation time scale might indicate different physical mechanisms that govern the streamer formation process. Streamers forming at very large time scales (*t*_*s*_ ~ *hrs*) have typically been reported in systems where formation of a biofilm occurs prior to streamer formation; referred herein as biofilm-mediated streamer formation^8^,^10^,^11^. To the best of our knowledge, a proper quantitative evaluation of small time scale streamer formation is yet to be reported. Furthermore, much of the literature on streamer formation in low Reynolds number conditions is relatively recent in the context of literature on biofilms and the physical basis of streamer formation remains an active area of research^9^,^20^,^21^.

In this study, we report our discovery of a new kind of bacterial streamer formation – these streamers do not appear from biofilms, rather they appear due to flow-induced deformation of the pre-formed bacterial flocs. We conduct our experiments in a microfluidic device composed of micropillars and containing solution laden with flocs of the bacterium *Pseudomonas flourescens*. We are able to optically probe the inception process of the streamers by embedding the bacterial flocs with 200 nm red fluorescent polystyrene beads that serve as tracers. We discover that fluid flow first advects the flocs some of which then get attached to the micropillars; subsequently, the hydrodynamic shear forces deform them into filamentous streamers. Interestingly, the formation timescale of this floc-mediated streamer formation is less than a second, which is in sharp contrast to the much larger timescale witnessed for biofilm-mediated streamers. Next, we find that the streamers are not purely elastic structures since we observed perceptible viscous behavior at time scales larger than its formation time scale but still far from clogging regime. Our methodology of using nanoscale fluorescent tracers, allows for the first time a direct quantification of the evolution of the streamer morphology. This method gives us valuable clues about the fundamental mechanism of floc-mediated streamer formation in contrast to the much more extensively studied biofilm-mediated streamer formation, which still remains a highly contentious problem.

## Results

### Initiation of floc-mediated streamers

**Figure 1:**
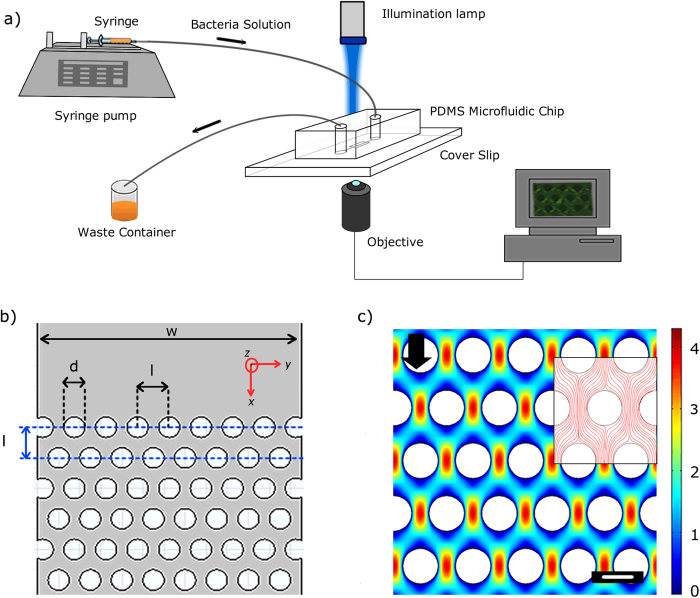
(**a**) A schematic of experimental set-up under pressure driven flow with constant volume flow rate (*Q*). (**b**) layout of staggered pattern porous media. Width (*W*) of porous zone is 625 μm. The distance between the center of pillars (*l*) and 2 rows of consecutive pillars is 75 μm. The diameter of the pillars and the height of the device (*h*_*c*_) both are 50 μm. (**c**) Computational fluid mechanical simulations demonstrating the non-dimensionalized velocity 
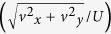
 contour of the flow in the porous section of the microchannel. *v*_*i*_ depicts fluid velocity in *i*-th direction. In our device *Q* = 15 μl/hr corresponds to flow speed of 1.3 × 10^−4^ m/s. The scale bar is 50 μm. (Inset) Streamlines for the same flow condition.

Our microfluidic device (Fig. 1a,b) consisted of a sequence of polydimethylsiloxane (PDMS) micropillars in a periodic staggered grid pattern. The micropillars had a diameter (*d*) of 50 μm and spaced 75 μm apart (*l*). The fluid flow rate (*Q*) was maintained at levels such that the resultant flow in our device was in the creeping flow regime (Reynolds number, *Re*, was *O* (10^−3^)). Numerical simulations provide the velocity profile inside the channel under these conditions. Figure 1c depicts the non-dimensionalized (with respect to *U* = *Q*/(*W* × *h*_*c*_) where *W* & *h*_*c*_ are channel width and height respectively) contour plot of magnitude of velocity and also streamlines (inset) in the device.

**Figure 2:**
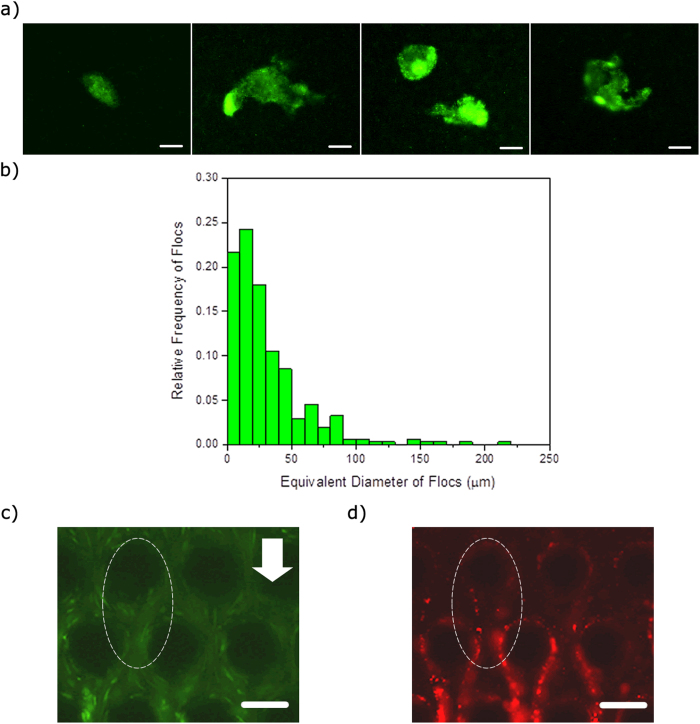
(**a**) Flocs of *P. fluorescens* bacteria after incubation at 30 °C for 2 days. The scale bars are 25 μm. (**b**) Relative frequency histogram of the flocs. The *x*-axis is equivalent diameter of the flocs in the reservoir. The median and mode for this relative frequency histogram are 21.28 and 22.23 μm, respectively. (**c**,**d**) Green and red fluorescent images, respectively, of the microchannel after injecting the bacteria with 200 nm fluorescent red polystyrene beads particles into it. The images were taken at the same time and place that was approximately at the middle of the channel height (*z* = 25 μm). The red fluorescent particles clearly seed and enable visualization of the streamer. Note the regions demarcated by dashed ellipses where bacteria (green) are not significant, but the streamer itself is easy visualized due to red particles seeding the EPS network. The scale bars are 50 μm.

We studied the behavior of the wild type (WT) strain of *Pseudomonas flourescens*, a bacteria that plays a vital role in maintaining plant physiology^22^. The genetically modified WT expressed green fluorescent protein (GFP) constitutively and hence was green fluorescent. Pre-formed biomass of *P. flourescens* in the form of bacterial flocs was utilized in our study (Fig. 2a). The bacterium was cultured in LB and M9 media (see Materials and Methods). While biomass/floc formation occurred in both media, flocs formed in M9 media were significantly smaller than the flocs produced in LB and they did not form streamers (see Fig. S2). This shows that growth conditions are also relevant factors; here we focus on flocs produced in LB media and defer investigation of growth conditions to a subsequent manuscript.

Bacterial flocs are EPS encapsulated aggregates of the bacteria that are dispersed in a liquid phase. These flocs were imaged first in quiescent media and their equivalent diameter was measured. For our system we observed a wide variation in the equivalent diameter. Their quantification was done through a relative frequency histogram, which shows that the mode for these flocs occurred at approximately 22 μm (Fig. 2b). These flocs were further mixed with 200 nm red fluorescent amine coated polystyrene (PS) particles and the mixture was allowed to flow through the microfluidic device. Two color imaging was performed for the system as the bacteria were green fluorescent and PS particles were red fluorescent. The red fluorescent beads were embedded in the EPS matrix of the flocs and thus provided clear visualization of the dynamics of these flocs (Fig. 2c,d). Such two-color visualization helps us overcome the difficulty in visualizing EPS, which is almost transparent under brightfield illumination.

**Figure 3:**
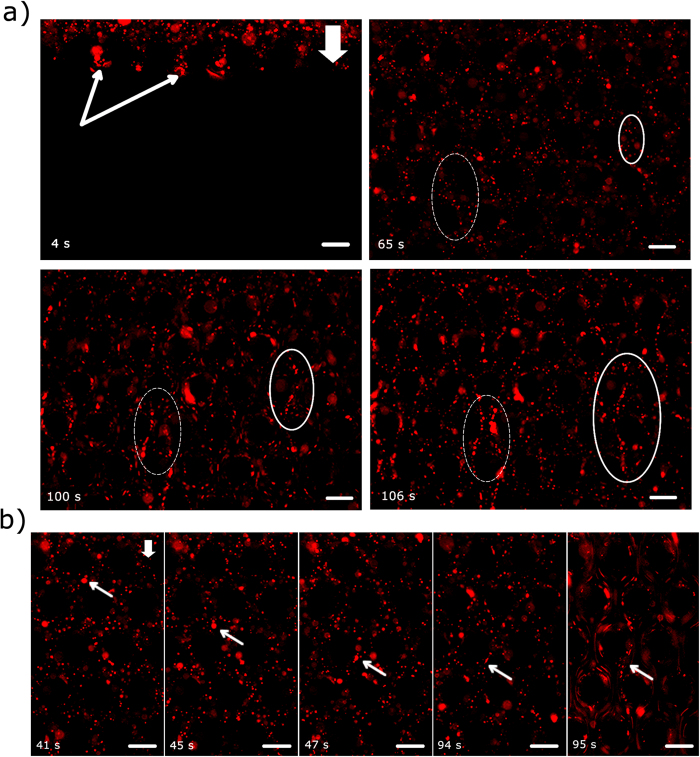
(**a**) Rapid streamer formation in a short time scale (a few seconds). The scale bars are 50 μm. See accompanying video. The images were taken approximately at the middle of the channel height (*z* = 25 μm). In the top-left image, the arrows demarcate the advancing fluid meniscus. The ellipses demarcate two regions where streamers form. (**b**) An arrow demarcates a floc, which is first advected through the channel and then is attached to a micropillar wall at *t* = 47 s and finally at 95 s a streamer is formed. Scale-bars are 50 μm.

The solution containing planktonic bacteria and bacterial flocs was flown through the device for several minutes and it was observed that streamer like structures formed within a few minutes of the initiation of the experiment (Fig. 3 & Supplementary Video 1). The bacterial flocs could be seen to attach to the micro-pillar posts and then deformed by fluid shear. A dashed ellipse marks the location of this event (Fig. 3a). Similarly, in the right hand side, another floc undergoes a similar process. It is interesting to note that the size distribution of flocs measured in quiescent media (Fig. 2a) is not reflected in the size of flocs that were observed to flow past the pillars (see Fig S1). For instance, although the mode of floc sizes was similar to the pillar diameter, such flocs were not observed in the flow past these pillars (see Supplementary information Fig. S1).

### Timescale and mechanism of floc-mediated streamer formation

**Figure 4:**
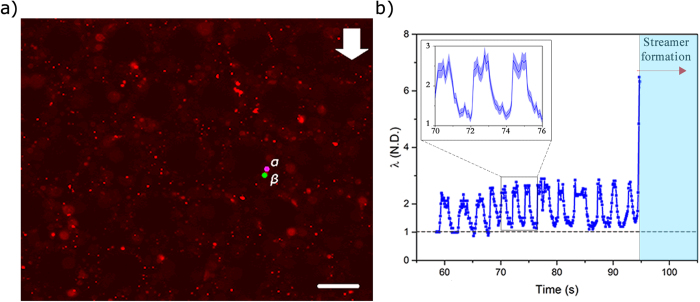
(**a**) Two points *α* and *β* are tracked as the fluid velocity fluctuates in the channel. These two points would later form a streamer. The scale bar is 50 μm. (**b**) Stretch ratio of the two points as a function of time during the initial filling period of the channel. Streamer formation region (right) also co-incides with the onset of steady flow. Black dashed line depicts λ = 1 (Inset) Stretch ratio for a smaller time segment. The colored envelope represents estimated error.

**Figure 5:**
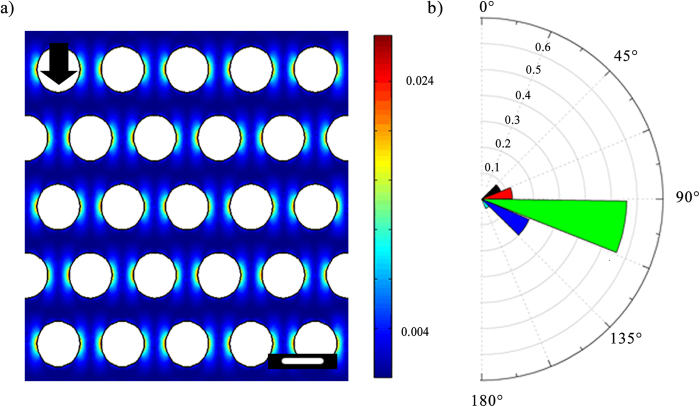
(**a**) Simulation results for the contour of the magnitude of the dimensionless shear stress 
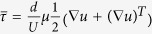
, *u* being the velocity field (**b**) Polar frequency histogram of where flocs attach on the pillars. Half of one pillar is considered. 0° represents upstream stagnation point and 180° represents downstream stagnation point.

The central result of our study is that the time-scale of formation of these floc-mediated streamers (*t*_*s*_) is very small (Fig. 3 & Supplementary Video 1). A close examination of Supplementary video 1 reveals that the floc in the right hand side of Fig. 3 undergoes large deformation at *t*_*s*_ < 1 sec. For instance, in the left hand side of Fig. 3, a floc approximately 3–3.5 μm in diameter undergoes very large deformation to form a streamer like structure. In Supplementary Video 1, a constant volume flow rate (or a constant *U*) is enforced by the syringe pump; however in the initial period when all pillars have not been wetted, *U* can have considerable oscillatory component in time^23^. Let us denote the time for this initial wetting as *t*_*wetting*_, which is observed to be about (~95 s) and taken to be the time when *U* becomes constant indicating the onset of completion of wetting. Thus at *t* > *t*_*wetting*_, we assume that wetting of all pillars is complete, and *U* becomes constant. For *t* < *t*_*wetting*_, i.e. when *U* has an oscillatory component in time, the response of the flocs to a temporally varying fluid shear at a spatial location can be seen clearly (Fig. 4 and Supplementary Video 1). Probing further, we track a set of closely placed particle couplets and measure the ratio of their separation along the streamer to their initial separation. This stretch ratio (*λ*) contains useful information regarding the material behavior of the streamer. To this end, a floc is chosen where embedded PS beads act as tracers and allow us to identify two closely situated points, *α* and *β*, and then these are tracked as a function of time (Fig. 4a). The point *α* is largely immobile due to its adhesion to the cylinder wall, while *β* is displaced by the fluid shear forces. As the fluid shear force scales with velocity 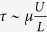, a time-periodic *U* results in a time-periodic *τ*. The distance between the two points in their initial (reference state) is denoted by *d***X** and in the current state by *d***x**. The axial stretch ratio defined as 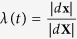 is plotted with respect to time in Fig. 4b. As expected, Fig. 4b indicates that, at *t* < *t*_*wetting*_, *λ(t)* oscillates between unity and a maximum value of approximately 3 (i.e. 200% engineering strain). This initial recoverable strain clearly indicates an elastic component of the streamer material. As the flow velocity increases, the streamer stretching begins to increase until, at the advent of steady flow, the floc is stretched into the slender geometry characteristic of streamer. After the initial wetting period (*t* > 95 s), the streamer becomes attached between two successive pillar walls indicating stretch of the order of seven (Fig. 4b). If a complete loss of material strength is assumed at this deformation, a lower limit of streamer formation time can be estimated from the background fluid velocity profile obtained from the simulations (Fig. 1c). Assuming an average transport speed of approximately *U* = 13 × 10^−5^ m/s, and transport distance as the inter-pillar separation length, of *l* = 75 × 10^−6^ m, we get 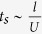 which comes to be approximately of the order of *t*_*s*_ is *O* (10^−1^) s. This time scale agrees well with our experiments. Furthermore, from the numerical simulations, the role of shear deformation in streamer formation is strongly suggested as well (see Fig. 5), since most of the streamers originate from regions corresponding to maximum shear stress.

**Figure 6:**
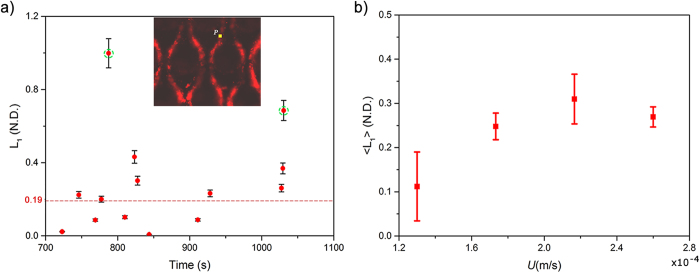
(**a**) Temporal variation of the non-dimensional principal velocity gradient (*L*_1_) of a fully formed streamer. The experimentally obtained values (red dots) show an approximately constant trend (dashed red line); two points demarcated through dashed green ellipses were neglected as outliers (Inset) A point ***P*** was tracked on a fully formed streamer for calculation of the velocity gradient. Here *U* = 1.3 × 10^−4^ m/s (**b**) Time-averaged principal velocity gradient (*L*_1_) of fully formed streamers as a function of flow speed (*U*).

Streamers eventually lead to catastrophic clogging of the device, such as that observed by Drescher *et al.*^8^. Interestingly however, the time period spanning the advent of streamer formation to final clogging is not marked by a sudden transition if closer look at the streamer behavior is taken. In this context, on an experimental time scale greater than the streamer formation time-scale (*t* > *t*_*s*_), but still far from clogging related change of overall velocity profile and streamer shape, there is a perceptible viscous component indicated by a creeping response of a material point of the streamer material (Supplementary Video 2). Quantification of this response is made possible by evaluation of the temporal response of the principal velocity gradient (strain rate) at a constant background velocity, which itself scales as the shear stress. This creeping response is well demonstrated by a temporal change in velocity gradients at the middle of the streamer. Although the velocity gradient variation for all streamers were found to be approximately constant in the time scale of scrutiny, for brevity we report the explicit temporal variation for a streamer only at velocity scale *U* of 1.3 × 10^−4^ m/s, Fig. 6a (see Materials and Methods). This indicates a steady creep regime under the flow conditions during the time scale of measurement. We further probe this behavior by plotting an averaged velocity gradient at a point for various flow rates, Fig. 6b. The figure clearly shows a nonlinear relationship between shear stress and strain rate and the slope, which is a rough estimation of the inverse of viscosity, shows a decreasing trend with stress. This suggests significant shear thickening component. Although the precise physical origin of this effect and the determination of the exact constitutive relationship to describe the inelasticity would need further experiments, this behavior is consistent with the structure of a typical biofilm and the relatively short period of observation. The film, which is itself a composite made up of suspended bacteria, flocs and aggregates in the EPS interact weakly with one another. However, as shear stress increases, these constituents come closer thereby increasing viscosity. Note that in time scales longer than the ones observed, several other complex phenomena such as mass aggregation and transition of inelasticity regimes would likely occur.

### Clogging action of the floc-mediated streamers

**Figure 7:**
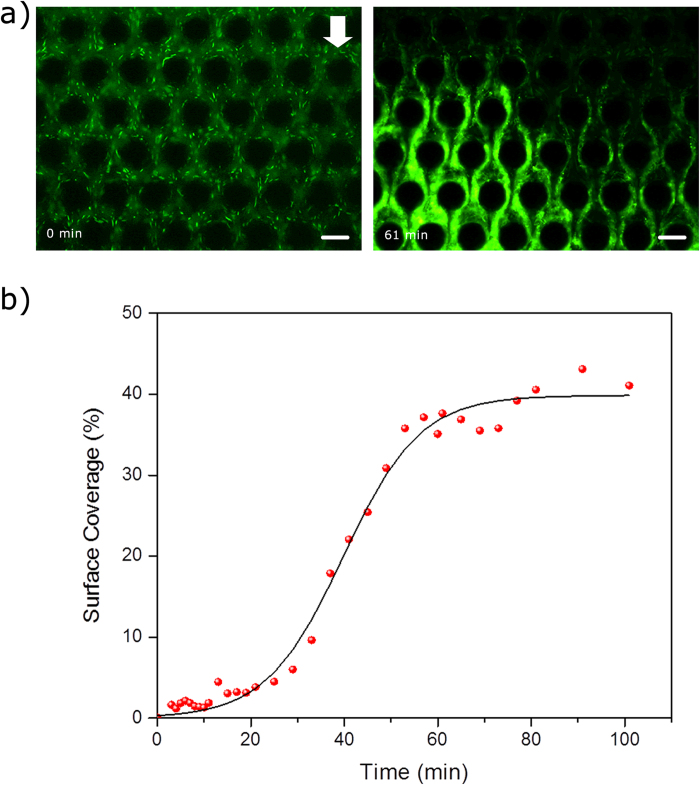
Rapid and catastrophic clogging of the channel by streamers. (**a**) Images of the channel at two time-points. The scale bars are 50 μm. (**b**) The graph shows a gradual increase of surface coverage from 2% (3 min) to 5% (25 min). Then, it has a dramatic surge from 5% (25 min) to 37% (57 min). After that the graph plateaus. Solid curve represents a sigmoidal curve fit to the data.

At a time-scale much greater than the streamer formation time-scale (*t* >> *t*_*s*_), a very different picture emerges. Streamer formation is a dynamic phenomenon where the streamer grows in width as it accrues additional mass from its surrounding fluid. Figure 7a shows that in approximately one hour after the beginning of the experiment a large part of the device is covered in biomass. This can be quantified by measuring the surface coverage by the biofilm as a function of time. Figure 7b shows that very quickly about 50% of the device is covered by the bacterial film. Thus clogging in this device can not only be catastrophic^8^ but also take place at a rapid clogging rate. The exponential increase in surface coverage can be explained by previously developed models for streamer growth as explained by Dreshcer *et al.*^8^ and later corrected by Das and Kumar^18^.

## Discussion

Here, using seeded particles, which allowed very precise quantification of the deformation of the biomass structures, we clearly demonstrate that pre-formed biomass, in the form of bacterial flocs, can lead to streamer formation through the process of large deformations, even when fluid flow in a system lies in the creeping flow regime. This is the first experimental observation, where the formation of a streamer has been demonstrated and we have shown that pre-formed biomass can lead to very rapid streamer formation time scales. Furthermore, particle tracking enabled us to conclude that the material behavior of the streamer can have both significant elastic as well as viscous component making them highly dynamic mechanical structures even in creeping flows. Finally, streamers cause not only catastrophic, but very rapid clogging of devices.

The exact material constitution of the bacterial communities is of great interest for a range of applications^9^. However, traditional material characterization techniques are impractical for *in situ* applications thus making the characterization of this system far more challenging. However, important material information is obtained by scrutinizing the response of the streamer due to fluidic loading by the background flow. The temporal behavior of the axial strain as quantified by *λ*(t), offers important insights into the mechanics of streamer formation. An interesting aspect of the streamer formation is the ability of the biomass to remain elastic under relatively large stretch ratios (engineering strain ~200%). This is typical of elastomeric materials. Such large strain elasticity is typically attributed to a molecular level ‘chain stretching’^24^. However, for the current material, it may also include straining of a more complex intermediate hierarchical microstructure well known in biofilm morphological literature^1^,^25^. This study would thus serve as an important motivation for such future extensions. As the flow develops fully, the shear stress on the incipient streamer increases significantly. This results in a significant increment of the shear stress causing the incipient structure to undergo even larger deformation after which the streamer extends between adjacent pillars and streamers are formed, see Fig. 3. The strain regime corresponding to this highly extended state (λ ~ 7), typically indicates substantial inelastic behavior. This is confirmed by observing a material point couple which move slowly through the streamer even when the background flow is constant (Fig. 6a,b). We assume that the measured velocity gradient at these deformations is almost entirely inelastic in nature.

**Figure 8:**
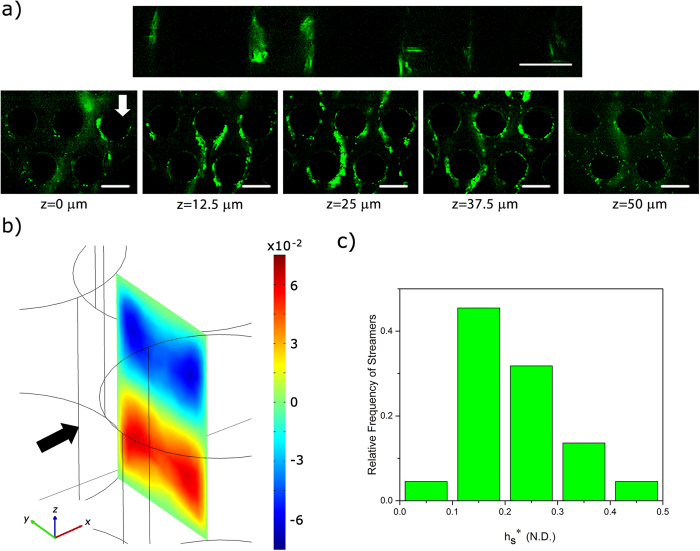
(**a**) Confocal sidebar shows the floc attachment through the height of the pillars. CLSM images of streamer formation at different heights in the chip. Such distribution of streamers across *z*-height is seen at other locations too. (**b**) Numerical simulation of the non-dimensional *z*-component of the velocity at a downstream location 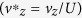 (arrow shows the direction of the flow). Secondary flows, in a direction transverse to the main flow, converge at the middle plane in the chip. (**c**) Histogram of streamer thickness as evaluated by CLSM after 30 mins of experimentation.

It is also important to note that the time-scale of streamer formation, as observed in the current investigation, is much smaller than both biological growth time-scales and visco-elastic relaxation time scales of biofilms^26^. This indicates that floc-driven streamer formation is physically distinct from biofilm-driven streamer formation^18^. In practical terms, the long time-lag observed in biofilm-driven streamer formation^8^,^10^,^11^ is not observed here, thus leading to very rapid streamer growth and clogging. We would also like to note that secondary flows seem to have little or no role in streamer location in our device. Rusconi *et al.*^10^ had credited secondary flows for the formation of a single streamer at the mid-height of their device. We investigated streamer distribution in the device along the *z*-axis using confocal laser scanning microscopy (CLSM) (Fig. 8a). In our device transverse secondary flows converging at the mid-plane of the device (similar to those seen by Rusconi *et al.*^10^) are also seen (Fig. 8b), but localization of streamers at *z* = *h*_*c*_/2 is not seen. In fact, we found that streamers were distributed at different heights in the device (Fig. 8a). The non-dimensional streamer thickness, h^*^ = *h*_*s*_/*h*_*c*_, where *h*_*s*_ is the dimensional streamer thickness, at time 30 mins is plotted in Fig. 8c.

Biofilm streamer formation remains an exciting frontier with several open ended unanswered questions. Here, we clearly demonstrate that pre-formed biomass, in the form of bacterial flocs, can lead to streamer formation through large deformations, even when fluid flow in a system lies in the creeping flow regime. This is the first direct experimental observation, where the formation of a streamer and its behavior in the intermediate time scale with respect to clogging has been demonstrated by using seeding particles for very precise quantification of the biomass structures. The key results of this work include demonstration of very rapid streamer formation and subsequent creep response of fully formed streamers. Finally, we show that streamer formation leads to rapid clogging of the device.

## Material and Methods

### Microchip fabrication

A 4″ silicon master mold was prepared by following the conventional photolithography process from the designed pattern. Then, by applying soft lithography processes and using the prepared master mold, the final device was fabricated from polydimethylsiloxane (PDMS, Sylgard 184, Dow Corning, NY, USA). Next, by exposing the cover slip and the PDMS stamp to oxygen plasma for 30 seconds, the PDMS stamp and the cover slip were bonded together to prepare the microchip. At the end, the microchip was annealed at 70 °C for 10 min to attain better sealing. The protocol is described in detail by Hassanpourfard *et al.*^14^.

### Bacterial culture

In this experiment, colonies of *Pseudomonas fluorescens* CHA0 (wild type) were grown on Luria-Bertani (LB) agar plate at 30 °C overnight. Next, one colony from an agar plate was inoculated into LB broth medium. One colony was also inoculated in M9 broth medium. To prepare the M9 broth, first, a 5× concentrated stock solution was prepared by stirring 56.4 g of power (containing the salt components 33.9 g/L Na_2_HPO_4_, 15 g/L KH_2_PO_4_, 5 g/L NH_4_Cl, and 2.5 g/L NaCl) in 1 L of water. This solution was autoclaved for 15 minutes at 121 °C in order to sterilize it. After cooling the 5× concentrated M9 stock, it was diluted to a 1× working solution by adding 200 ml of the 5× M9 stock to 700 ml sterile water. Afterwards, 2 ml of sterile 1 M MgSO_4_, 0.1 ml of sterile 1 M CaCl_2_ and 20 ml of 20% glucose were added. Then, the solution was adjusted to have a final volume of 1 L by adding sterilized distilled water (the final glucose concentration was 0.4%). The strain of bacteria here is green fluorescent as they express green fluorescent protein (GFP) constitutively. To stimulate floc formation in the bacterial solution, the bacterial solution was kept in an incubator (New Brunswick Scientific Co., NJ) at 30 °C for about 48 h. Longer incubation duration leads to nutrient depletion and subsequent floc formation^27^. Here in the LB medium, the OD_600_ for the bacterial culture was approximately 1.7 after 48 hours and for the M9 medium under the same conditions the OD_600_ was approximately 0.7.

### Microscopy

The microfluidic chip was placed on the stage of an inverted optical microscope (Nikon Eclipse Ti). The bacteria solution was injected continuously into the microchip by using a syringe pump (Harvard Apparatus, MA, USA). The temperature of microchip was set at 30 °C by the aid of an on-stage microscope incubator (Pathologic Devices, Inc., MD, USA). Particle tracking was performed using the object tracking module in Nikon NIS-Element AR software interface. Effective surface area calculations were also performed using the same software. Error for stretch ratio calculation was estimated to be approximately 8%. Surface coverage percentage was calculated by defining a threshold value for green color intensity. If the green color intensity of a pixel was above the threshold value then corresponding pixel was counted. Next, we calculated the surface coverage percentage by dividing the counted pixels to the total number of pixels in the image (except area covered by the pillars) and multiplying by 100. This procedure is the same as that outlined in a previous publication by Kumar *et al.*^28^. Furthermore, the streamer’s location in different *z* directions was investigated by capturing *z*-stack images using a confocal laser scanning microscope (CLSM) (Carl Zeiss, Inc.,NY, USA).

### Computational fluid mechanical (numerical) simulations

We performed fluid mechanical simulations using the commercial package Comsol Multiphysics® to simulate the device flow. In this simulation, we assumed that the fluid going through the channel has the same properties as water at 30 °C and pressure. To describe the fluid flow in the channel the incompressible Navier-Stokes and continuity equations were used. The inlet velocity was calculated according to the flow rate (here 15 μl/h). The no-slip and the no penetration boundary condition were imposed on the walls and a constant atmospheric pressure was imposed at the channel outlet, respectively. Mesh density was increased until no mesh dependency was observed in the solution. Velocity is non-dimensionalized with respect to the velocity scale, *U* = *Q*/(*W* × *h*_*c*_), where *Q* is the volume flow rate (imposed by the syringe pump).

### Calculation Velocity Gradient

We tracked a particle **P** which moves in a fixed Eulerian grid from *P*_0_*(x*_0_*, y*_0_) to *P*_1_*(x*_1_*, y*_1_) in time *δt*_*1*_ and then further moves to *P*_2_*(x*_2_*, y*_2_) in another time *δt*_*2*_.

The normalized velocity gradient at *P*_*0*_ is then given by forward-difference discretization:


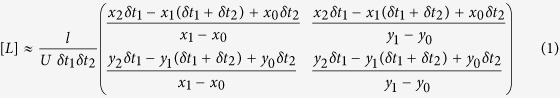


Two eigenvalues of [*L*] corresponding to two principal eigenvectors were found. One of the eigenvectors was found to be nearly aligned with the orientation of the streamer and the eigenvalue corresponding to the other eigenvector was vanishingly small as expected. Thus for the purpose of this work, we denote the larger eigenvalue (which is aligned with the streamer orientation) of the velocity gradient tensor as the principal velocity gradient, *L*_1_. Note that this principal velocity gradient component is assumed to be mostly inelastic in the time frame considered with elastic and rotational components assumed negligible.

## Additional Information

**How to cite this article**: Hassanpourfard, M. *et al.* Bacterial floc mediated rapid streamer formation in creeping flows. *Sci. Rep.* **5**, 13070; doi: 10.1038/srep13070 (2015).

## Supplementary Material

Supplementary Information

Supplementary Video 1

Supplementary Video 2

## Figures and Tables

**Table 1:** Time scale of streamer formation from different experiments.

**Bacteria**	**Streamer formation time scale (hr)**	**Ref.**	
*Pseudomonas aeruginosa*	6–7	Rusconi *et al.*^10^	
*Pseudomonas aeruginosa*	18	Rusconi *et al.*^29^	
*Pseudomonas fluorescens*	few hours	Valiei *et al.*^11^	
*Pseudomonas aeruginosa*	50	Drescher *et al.*^8^	
*Staphylococcus epidermis*	6	Weaver *et al.*^30^	
*E. Coli*	0.5	Yazdi and Ardekani^16^	
*Pseudomonas fluorescens*	~10^−3^ (i.e. a few seconds)	Present work	

## References

[b1] Flemming H. C. & Wingender J. The biofilm matrix. Nat Rev Microbiol 8, 623–633, 10.1038/Nrmicro2415 (2010). 20676145

[b2] Wingender J., Neu T. R. & Flemming H.-C. in Microbial extracellular polymeric substances 1–19 (Springer, 1999).

[b3] Wu C., Lim J. Y., Fuller G. G. & Cegelski L. Quantitative Analysis of Amyloid-Integrated Biofilms Formed by Uropathogenic Escherichia coli at the Air-Liquid Interface. Biophys. J. 103, 464–471, 10.1016/j.bpj.2012.06.049 (2012). 22947862 PMC3414876

[b4] Whitfield C. Bacterial extracellular polysaccharides. Can J Microbiol 34, 415–420 (1988). 3052752 10.1139/m88-073

[b5] Flemming H. C., Neu T. R. & Wozniak D. J. The EPS matrix: The “House of Biofilm cells”. J Bacteriol 189, 7945–7947, 10.1128/Jb.00858-07 (2007). 17675377 PMC2168682

[b6] More T. T., Yadav J. S. S., Yan S., Tyagi R. D. & Surampalli R. Y. Extracellular polymeric substances of bacteria and their potential environmental applications. J Environ Manage 144, 1–25, 10.1016/j.jenvman.2014.05.010 (2014). 24907407

[b7] Friedman B. A., Dugan P. R., Pfister R. M. & Remsen C. C. Structure of Exocellular Polymers and Their Relationship to Bacterial Flocculation. J Bacteriol 98, 1328-& (1969). 5788706 10.1128/jb.98.3.1328-1334.1969 PMC315330

[b8] Drescher K., Shen Y., Bassler B. L. & Stone H. A. Biofilm streamers cause catastrophic disruption of flow with consequences for environmental and medical systems. P Natl Acad Sci USA 110, 4345–4350, 10.1073/pnas.1300321110 (2013). PMC3600445 23401501

[b9] Karimi A., Karig D., Kumar A. & Ardekani A. Interplay of physical mechanisms and biofilm processes: review of microfluidic methods. Lab Chip 15, 23–42, 10.1039/C4LC01095G (2015). 25385289 PMC4261921

[b10] Rusconi R., Lecuyer S., Guglielmini L. & Stone H. A. Laminar flow around corners triggers the formation of biofilm streamers. J R Soc Interface 7, 1293–1299, 10.1098/rsif.2010.0096 (2010). 20356880 PMC2894892

[b11] Valiei A., Kumar A., Mukherjee P. P., Liu Y. & Thundat T. A web of streamers: biofilm formation in a porous microfluidic device. Lab Chip 12, 5133–5137, 10.1039/C2lc40815e (2012). 23123600

[b12] Stoodley P., Cargo R., Rupp C. J., Wilson S. & Klapper I. Biofilm material properties as related to shear-induced deformation and detachment phenomena. J. Ind. Microbiol. Biot. 29, 361–367 (2002). 10.1038/sj.jim.7000282 12483479

[b13] Stoodley P., Lewandowski Z., Boyle J. D. & Lappin-Scott H. M. Oscillation characteristics of biofilm streamers in turbulent flowing water as related to drag and pressure drop. Biotechnol Bioeng 57, 536–544 (1998). 10099232 10.1002/(sici)1097-0290(19980305)57:5<536::aid-bit5>3.0.co;2-h

[b14] Hassanpourfard M. *et al.* Protocol for Biofilm Streamer Formation in a Microfluidic Device with Micro-pillars. JoVE (Journal of Visualized Experiments), e51732–e51732 (2014). 10.3791/51732 PMC4758759 25178035

[b15] Marty A., Roques C., Causserand C. & Bacchin P. Formation of bacterial streamers during filtration in microfluidic systems. Biofouling 28, 551–562, 10.1080/08927014.2012.695351 (2012). 22686836

[b16] Yazdi S. & Ardekani A. M. Bacterial aggregation and biofilm formation in a vortical flow. Biomicrofluidics 6, 10.1063/1.4771407 (2012). PMC3555698 24339847

[b17] Marty A., Causserand C., Roques C. & Bacchin P. Impact of tortuous flow on bacteria streamer development in microfluidic system during filtration. Biomicrofluidics 8, 014105 (2014). 24753726 10.1063/1.4863724 PMC3977864

[b18] Das S. & Kumar A. Formation and post-formation dynamics of bacterial biofilm streamers as highly viscous liquid jets. Scientific reports 4, 7126, 10.1038/srep07126 (2014). 25410423 PMC4237988

[b19] Kim M. K., Drescher K., Pak O. S., Bassler B. L. & Stone H. A. Filaments in curved streamlines: rapid formation of Staphylococcus aureus biofilm streamers. New J Phys 16, 065024, 10.1088/1367-2630/16/6/065024 (2014). PMC4255984 25484614

[b20] Hol F. J. H. & Dekker C. Zooming in to see the bigger picture: Microfluidic and nanofabrication tools to study bacteria. Science 346, 438-+, 10.1126/Science.1251821 (2014). 25342809

[b21] Rusconi R., Garren M. & Stocker R. Microfluidics Expanding the Frontiers of Microbial Ecology. Annual Review of Biophysics 43, 65–91, 10.1146/annurev-biophys-051013-022916 (2014). PMC4076152 24773019

[b22] Mercado-Blanco J. & Bakker P. A. Interactions between plants and beneficial Pseudomonas spp.: exploiting bacterial traits for crop protection. Antonie van Leeuwenhoek 92, 367–389, 10.1007/s10482-007-9167-1 (2007). 17588129

[b23] Chen H.-H., Shi J. & Chen C.-L. Wetting dynamics of multiscaled structures. Appl. Phys. Lett. 103, 171601 (2013).

[b24] Arruda E. M. & Boyce M. C. A three-dimensional constitutive model for the large stretch behavior of rubber elastic materials. J Mech Phys Solids 41, 389–412 (1993).

[b25] De Beer D., Stoodley P., Roe F. & Lewandowski Z. Effects of biofilm structures on oxygen distribution and mass transport. Biotechnol Bioeng 43, 1131–1138 (1994). 18615526 10.1002/bit.260431118

[b26] Shaw T., Winston M., Rupp C. J., Klapper I. & Stoodley P. Commonality of elastic relaxation times in biofilms. Phys Rev Lett 93, 10.1103/Physrevlett.93.098102 (2004). 15447143

[b27] De Schryver P., Crab R., Defoirdt T., Boon N. & Verstraete W. The basics of bio-flocs technology: The added value for aquaculture. Aquaculture 277, 125–137, 10.1016/j.aquaculture.2008.02.019 (2008).

[b28] Kumar A. *et al.* Microscale confinement features can affect biofilm formation. Microfluid Nanofluid 14, 895–902, 10.1007/s10404-012-1120-6 (2013).

[b29] Rusconi R., Lecuyer S., Autrusson N., Guglielmini L. & Stone H. A. Secondary flow as a mechanism for the formation of biofilm streamers. Biophys J 100, 1392–1399, 10.1016/j.bpj.2011.01.065 (2011). 21402020 PMC3059581

[b30] Weaver W. M., Milisavljevic V., Miller J. F. & Di Carlo D. Fluid flow induces biofilm formation in Staphylococcus epidermidis polysaccharide intracellular adhesin-positive clinical isolates. Appl Environ Microbiol 78, 5890–5896, 10.1128/AEM.01139-12 (2012). 22706049 PMC3406141

