# Supplementary material for: Bacterial floc mediated rapid streamer formation in creeping flows
Source: Sci Rep. 2015 Aug 17;5:13070. doi: 10.1038/srep13070 (PMC4538384; doi:10.1038/srep13070)
Supplement: Supplementary Information [file srep13070-s1.pdf]

Supplementary Information for

Bacterial floc mediated rapid streamer formation in creeping flows

Mahtab Hassanpourfard[a], Zahra Nikakhtari[b], Ranajay Ghosh[c], Siddhartha Das[d], Thomas Thundat[a], Yang Liu[e], Aloke Kumar[b]*

[a]Department of Chemical and Materials Engineering, University of Alberta, Edmonton, Canada

[b]Department of Mechanical Engineering, University of Alberta, Edmonton, Canada

[c]Department of Mechanical and Industrial Engineering, Northeastern University, Boston MA 02115, USA

[d] Department of Mechanical Engineering, University of Maryland, College Park, MD 20742, USA

[e]Department of Civil and Environmental Engineering, University of Alberta, Edmonton, Canada

*Email for Correspondence: aloke.kumar@ualberta.ca

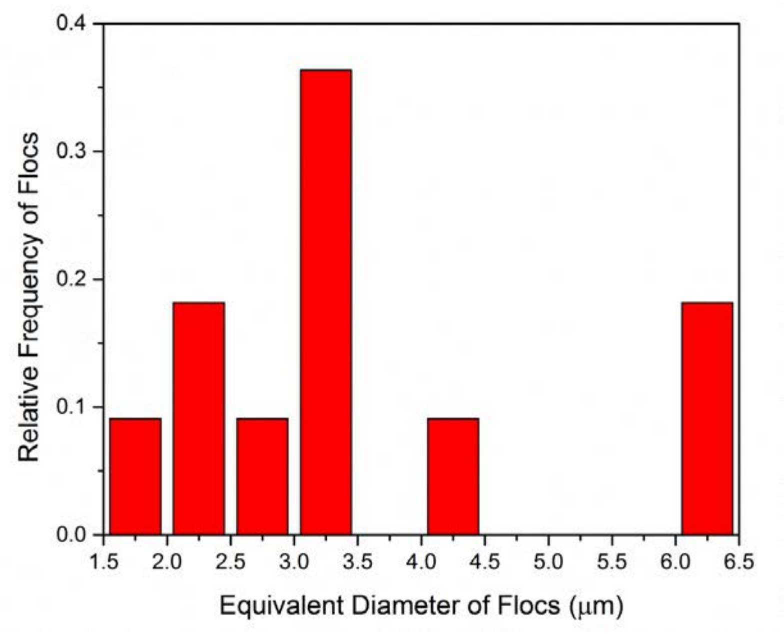

Figure S1: Relative frequency of flocs sizes entering the microfluidic channel.

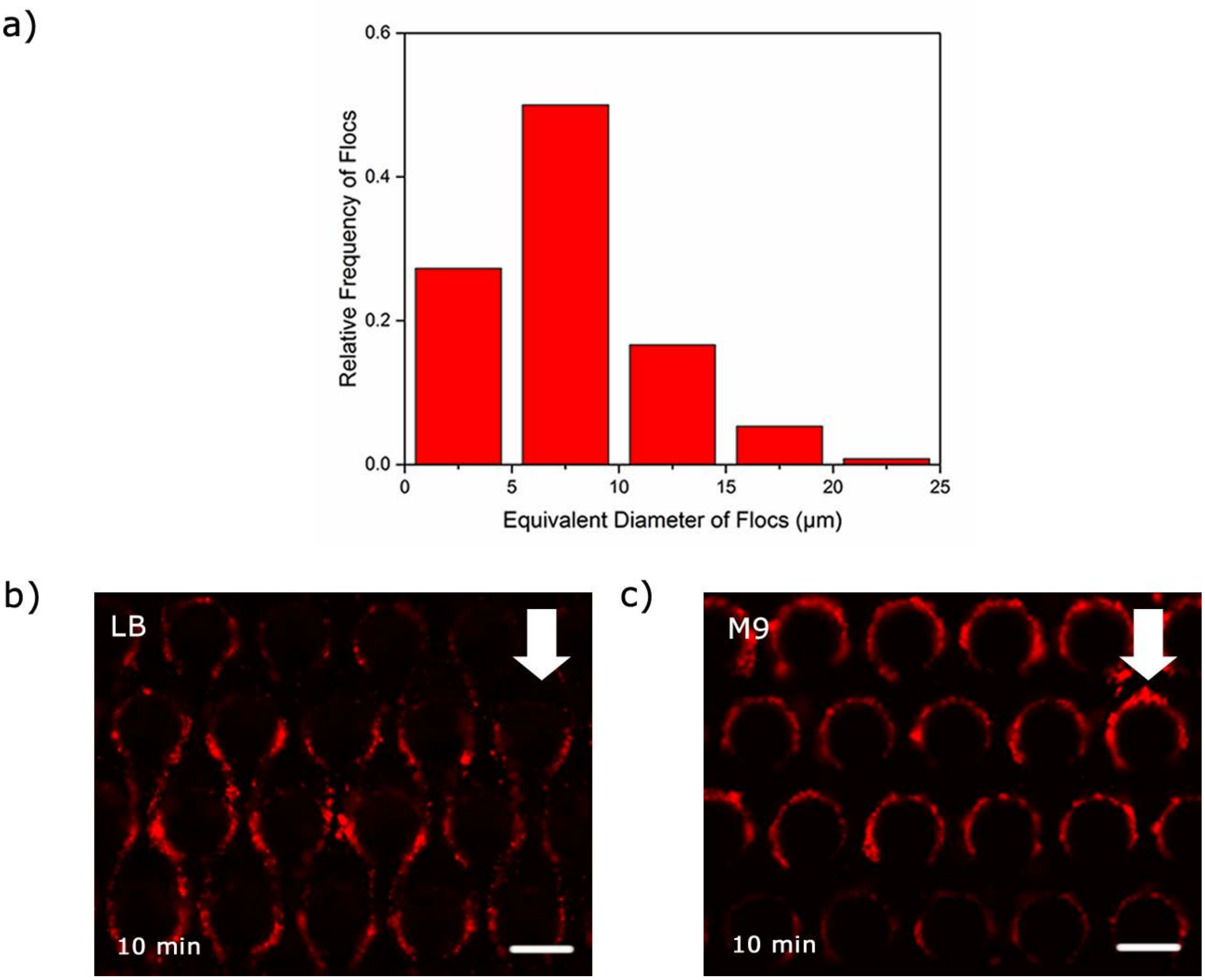

Figure S2: (a) Relative frequency histogram of the flocs cultured in M9 media. The median and mode for this relative frequency histogram are 6.78 and 5.85 µm, respectively (b) & (c) Comparison of streamer formation in LB and M9 media. Streamers did not form in M9 media at short-time scales (~ few minutes) as compared to LB media.

Supplementary Videos

Supplementary Video 1: Video shows formation of streamers. Video runs at 2X i.e. 1 second of video time represents 2 seconds of experimental time. Scale bar represents 50 µm.

Supplementary Video 2: Video shows the large time scale movement of the streamer. A timer (hr:min:secs:miliseconds) depicts the experimental time. Note that the video has been sped up 32x. Scale bar represents 50 µm.
